# Glucocorticoids influence versican and chondroitin sulphate proteoglycan levels in the fetal sheep lung

**DOI:** 10.1186/s12931-018-0854-4

**Published:** 2018-08-20

**Authors:** Annie R. A. McDougall, Amanda J. Fosang, Jessica Faggian, Megan J. Wallace, Kelly J. Crossley, Timothy J. Cole, Stuart B. Hooper

**Affiliations:** 1 The Ritchie Centre, The Hudson Institute of Medical Research, 27-31 Wright Street, Clayton, VIC 3168 Australia; 2 Arthritis Research Group, Department of Pediatrics, University of Melbourne and Murdoch Children’s Research Institute, Royal Children’s Hospital, Parkville, Victoria, 3052 Australia; 3 Department of Obstetrics and Gynaecology, Monash University, Melbourne, VIC 3800 Australia; 4 Department of Biochemistry and Molecular Biology, Monash University, Melbourne, VIC 3800 Australia

**Keywords:** Cortisol, Betamethasone, Lung development, Extracellular matrix, Adrenalectomy

## Abstract

**Background:**

Prenatal glucocorticoid treatment decreases alveolar tissue volumes and facilitates fetal lung maturation, however the mechanisms responsible are largely unknown. This study examines whether changes in versican levels or sulphation patterns of chondroitin sulphate (CS) side chains, are associated with glucocorticoid-induced reductions in peri-alveolar tissue volumes.

**Methods:**

Lung tissue was collected from 1) fetal sheep at 131 ± 0.1 days gestational age (GA) infused with cortisol (122-131d GA) to prematurely induce a pre-parturient-like rise in circulating cortisol, 2) fetal sheep at 143d GA bilaterally adrenalectomised (ADX) at 112d GA to remove endogenous cortisol and 3) fetal sheep at 124d GA in which bolus doses (2 × 11.4 mg) of betamethasone were administered to the pregnant ewe. The level and distribution of versican and CS glycosaminoglycans (GAG) were determined using immunohistochemistry (IHC). Fluorophore assisted carbohydrate electrophoresis (FACE) was used to determine changes in CS sulphation patterns.

**Results:**

Cortisol infusion significantly decreased chondrotin-6-sulphate levels (C-6-S) to 16.4 ± 0.7 AU, compared with saline-infused fetuses (18.9 ± 0.7 AU: *p* = 0.04) but did not significantly alter the level of versican or chondroitin-4-sulphate (C-4-S). ADX significantly increased the level of C-4-S (28.2 ± 2.2 AU), compared with sham-operated fetuses (17.8 ± 2.0 AU; *p* = 0.006) without altering versican or C-6-S levels. Betamethasone significantly decreased versican, C-4-S and C-6-S in the fetal sheep lung (19.2 ± 0.9 AU, 24.9 ± 1.4 AU and 23.2 ± 1.0 AU, respectively), compared with saline-exposed fetuses (24.3 ± 0.4 AU, *p* = 0.0004; 33.3±0.6 AU, *p* = 0.0003; 29.8±1.3 AU, 0.03, respectively).

**Conclusions:**

These results indicate that glucocorticoids alter versican levels and CS side chain microstructure in alveolar lung tissue. Betamethasone appears to have a greater impact on versican and CS side chains than cortisol.

## Background

Antenatal synthetic corticosteroids greatly improve postnatal lung function and markedly reduce the risk of respiratory distress syndrome in preterm infants [1] by facilitating maturation of the distal lung parenchyma and increasing lung tissue compliance [2–4]. The maturational changes include increased surfactant synthesis [5], reduced alveolar wall thickness [6–9] and reduced interstitial tissue volumes [9] that replicate the developmental changes in lung structure that occur during late gestation. As these reductions in lung tissue volumes are also closely associated with a reduction in versican levels, an extracellular proteoglycan involved in tissue volume regulation, it has been suggested that reduced versican levels mediate the age-related reduction in lung tissue volumes [10]. More recently, studies in mice lacking the glucocorticoid receptor demonstrate that the reduction in peri-alveolar tissue volumes induced by glucocorticoids involves alterations in cell proliferation [11, 12] and remodelling of the extracellular matrix (ECM) [13]. While, the mechanisms and specific ECM components involved remain largely unknown we have recently shown that versican is a target of glucocorticoid signalling in the developing lung [11]. Furthermore, it is now clear that versican can regulate cell adhesion, survival, proliferation, migration and ECM assembly [14], making it an ideal candidate for mediating some of the corticosteroid induced effects on fetal lung structure.

Versican is the most abundant chondroitin sulphate (CS) proteoglycan in the lung. CS proteoglycans consist of a protein core with one or more covalently bound glycosaminoglycans (GAGs) (reviewed in [15]). CS GAGs are linear polymers composed of repeating disaccharide units of glucuronic acid (GlcUA) and N-acetylgalactosamine (GalNAc). The CS disaccharides, [− 4)GlcA(β1–3)GalNAc(β1-] may be sulphated at C_4_ and/or C_6_ of GalNAc and at C_2_ of GlcUA. Variations in the position and degree of sulphation within GAGs create substantial structural and functional diversity for CS proteoglycans. In addition, some CS proteoglycans (such as versican) are alternatively spliced to form up to four versican isoforms, creating variants with differing glycosaminoglycan attachment domains, further contributing to the functional diversity. The high anionic charge density of large proteoglycans, such as versican attracts mobile counter ions to maintain electroneutrality and, in turn, generates the osmotic swelling pressure that regulates interstitial tissue hydration and solute permeability, as well as influencing the viscoelastic properties of tissue. Thus, alterations in the structural properties of versican, leading to changes in its anionic charge density, may regulate tissue volumes and viscoelastic properties of the distal airways in the developing lung. Indeed, in addition to showing that versican levels are closely associated with changes in lung tissue volumes during development, we have also shown marked changes in the microstructure of CS side chains in lung tissue during development [10]. The mechanisms responsible for these changes in versican levels are unknown, although it is possible that endogenous cortisol, which is known to mature the fetal lung in late gestation, may be involved [11].

Our aim was to investigate the effect of endogenous and synthetic corticosteroids on versican deposition and mRNA levels as well as sulphation patterns of CS side chains, and other CS-proteoglycans in the fetal lung. In view of the direct relationship between reductions in peri-alveolar lung tissue volumes and reductions in the level of versican and CS GAGs [10], we hypothesised that corticosteroid-induced reductions in peri-alveolar tissue volumes result from either a decrease in versican content (due to changes in mRNA levels or protein deposition), or changes in the degree or pattern of sulphation on CS GAG chains, leading to a reduction in charge density and osmotic activity. To test these hypotheses, we examined versican expression and content as well as CS sulphation patterns in fetal sheep lung tissue exposed to three different glucocorticoid treatments; 1) fetuses infused over 9 days with increasing concentrations of cortisol, designed to mimic the pre-parturient increase in fetal plasma cortisol concentrations, 2) bi-lateral adrenalectomised fetuses, that lack an endogenous source of fetal cortisol, and 3) fetuses exposed to antenatal betamethasone treatment, administered to the ewes.

## Methods

### Animals

#### Cortisol-treated fetuses

Fetal lung tissues from cortisol-infused fetuses were obtained from previously published studies [16]. Cortisol infused fetuses (*n* = 5) received increasing doses of cortisol (hydrocortisone sodium succinate, Solu Cortef; Upjohn) for a period of 9 days; cortisol was dissolved in heparinised saline and infused (1.2 ml/h) into the jugular vein at 1.5 mg/day on 122-123d GA, 2.5 mg/day on 124-125d GA, 3.0 mg/day on 126-127d GA, 3.5 mg/day on 128-129d GA and 4.0 mg/day on 130-131d GA. This dose induced a precocious preparturient-like rise in circulating cortisol levels [16]. Saline-infused control fetuses (*n* = 5) received an infusion of heparinised saline (1.2 ml/h) from 122-131d GA. Fetal blood samples (~ 2 mL) were collected every 2–3 days to measure plasma cortisol concentrations. All ewes and fetuses were humanely killed at 131d GA (early alveolar stage; alveolar stage begins 36 weeks GA in humans) and fetal lung tissue collected.

#### Adrenalectomised fetuses

Aseptic surgery was carried out on pregnant Border Leicester x Merino ewes at 112 ± 3d GA for implantation of fetal and maternal vascular catheters. Fetuses were then either bi-laterally adrenalectomised (ADX; *n* = 6) as described previously [17] or sham-operated (controls; n = 6) to expose but not remove both adrenal glands. Fetal well-being was assessed every second day via measurements of fetal arterial blood pH, partial pressure of CO_2_, partial pressure of O_2_, and percent saturation of O_2_. Fetal blood samples were collected every 5 days from 120d GA to measure plasma cortisol concentrations and ensure that the pre-parturient increase in fetal plasma cortisol concentrations did not occur. Ewes and fetuses were humanely killed at 143 ± 1d GA (alveolar stage; equivalent to ~ 39 weeks GA in humans) and fetal lung tissue collected.

#### Betamethasone exposed fetuses

Unoperated ewes (*n* = 6) were administered a bolus intramuscular dose of betamethasone (11.4 mg: 5.7 mg/ml: Celestone Chronodose, Schering-Plough, Australia) at 36 h and 24 h prior to post-mortem at 124 d GA. Ewes bearing control fetuses (*n* = 6) were administered the equivalent volume of saline at the same gestational ages and at the same time interval as per betamethasone-treated ewes. All ewes and fetuses were humanely killed at 124d GA (late canalicular/early alveolar stage; canalicular stage last until 26 weeks GA in humans) and fetal lung tissue collected.

### Tissue collection

Ewes and fetuses were humanely killed using an overdose of pentobarbitone sodium (6.5 g *i.v.)* administered to the ewe. Fetuses were removed and the fetal body and organ weights recorded. The ADX fetuses were examined for adrenal tissue regrowth. The left main bronchus of the fetal lung was ligated and small portions of the parenchymal tissue distal to this ligature were collected, frozen in liquid nitrogen and stored at − 70°C for biochemical analyses. The right lung was pressure fixed at 20 cmH_2_O and then processed for histological analyses.

### Tissue analysis

#### Tissue/airspace volume

Following fixation, the right lung was separated into the upper, middle and lower lobes. Each lobe was cut into 5 mm slices before a minimum of 9 tissue slices (at least 3 slices per lobe) were randomly chosen from each lung. A small portion (approximately 2cm^2^ × 5 mm thick) was randomly selected from each slice and embedded into paraffin blocks. At least 5 blocks per animal were selected at random and 5 μm tissue sections were cut, stained with Haematoxylin and Eosin and viewed under the light microscope. Using a minimum of 2 fields of view per block (10 fields of view per animal), the percentage of airspace and tissue space volumes were calculated using a point counting technique as previously described [18].

#### Immunohistochemistry

Versican protein was localised as previously described [10]. The primary antibody used to detect the versican core protein was a rabbit polyclonal antibody (gift from Professor Dick Heinegard, Lund, Sweden). The primary antibodies used to detect chondroitin-4 sulphate (C-4-S) and chondroitin-6-sulphate (C-6-S) were the mouse monoclonal antibodies 2030 and 2035, respectively (Chemicon International). Briefly, 10μm sections cut from randomly selected frozen fetal lung tissue were mounted on glass slides and fixed for 20 min in 4% paraformaldehyde. The slides were digested with 0.02 U chondroitinase ABC (in 0.1 M Tris-HCl, pH 8, 30 m Na Acetate, 1 mM EDTA; containing E64, AEBSF & pepstatin protease inhibitors; Seikagaku, Japan) for 60 min at 37°C. Sections were then , incubated in blocking/permeabilization buffer (3% normal goat serum; 0.1% TritonX-100 in PBS) in a humidity chamber for 30 min at 37 °C. Sections were incubated with the primary antibody overnight at 4 °C, followed by incubation with secondary antibody (2% normal goat serum; 1:700 dilution of goat anti-rabbit serum or 1:800 goat anti-mouse serum in PBS, depending on the primary antibody) for 1 h at room temperature. The process was repeated with a second primary antibody and a second (different from the first) secondary antibody and slides were mounted with Vectashield (Vector laboratories).

**Fig. 1:**
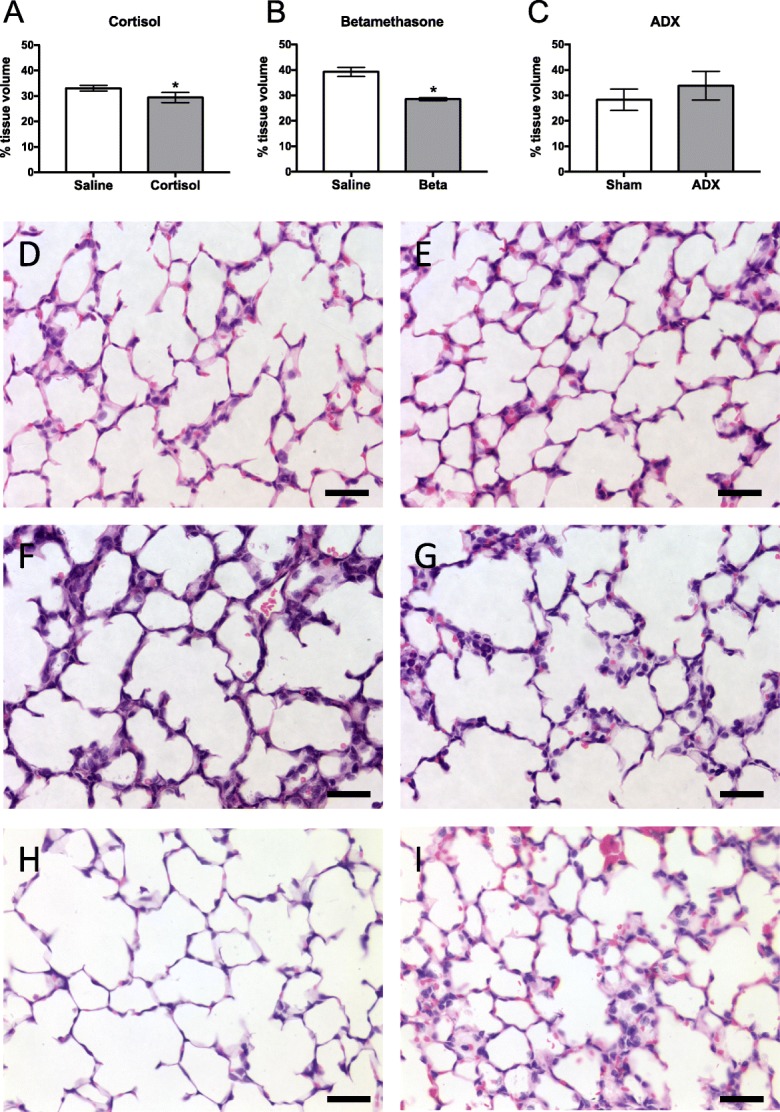
Mean ± SD fractional tissue volume in (**a**) cortisol-treated (*n* = 6) and saline-treated (*n* = 5), (**b**) betamethasone-treated (n = 6) and saline-treated (n = 6), and (**c**) ADX- (n = 6) and sham-operated (*n* = 6) sheep lungs. Representative images of H&E stained lung sections from (**d**) saline- and (**e**) cortisol-treated sheep, (**f**) saline- and (**g**) betamethasone-treated sheep, and (**h**) sham- and (**i**) ADX-operated sheep. *p < 0.05 compared to controls. Scale = 25μm

Immunofluorescent markers were visualised and digital images captured using a Nikon Eclipse 80i laser scanning microscope system. Fluorophores were excited specifically using 488 or 561 nm lasers and fluorescence detected using 515/30 nm and 605/75 nm filters respectively. At least 2 sections (from different regions of the lungs) were viewed from each animal and multiple fields of view (> 3) were analysed per section; care was taken to avoid fields of view that included major airways and blood vessels. Digital images were captured using the × 40 objective and the mean intensity of staining (measured in arbitrary units (AU), to provide a relative change between samples from individual experiments) was quantified using Image Pro Plus image analysis software (Media Cybernetics, USA). To correct for the reduction in tissue volume between treatment and experimental groups, the mean intensity of versican staining for each individual fetus was adjusted for the tissue fraction calculated for that animal (Fig. 1), to give the relative versican level per unit volume of lung tissue. Imaging and analysis of immunoreactivity was performed in a single day using identical parameters (laser strength, exposure time) to maintain consistency and eliminate error, for each antibody.

#### Versican gene expression

The mRNA levels of versican in fetal lung tissue were measured using realtime qPCR as previously described [19]. Briefly, total RNA was isolated from fetal lungs using the RNeasy midi RNA extraction kit (Qiagen, Australia). Total RNA (1 μg) was then reverse transcribed using Superscript® III Reverse Transcriptase (Invitrogen, Australia). Relative levels of *Vcan* mRNA (the gene for versican; forward primer 5’-TGTTTGTGAATCGTGTGGGC-3′; reverse primer 5’-GCTGTCTGGTTGGTTTGGTC-3′; primers designed to detect all splice variants of *Vcan*) was quantified by real-time qPCR with the housekeeping gene *ribosomal protein 29 (Rps29;* forward primer 5’-CAGGGTTCTCGCTCTTGC-3′; reverse primer 5’-ACTGGCGGCACATATTGAG-3′*)* using the ΔCt method of analysis and expressed as a fold change relative to the mean level in the relative control group.

#### CS analysis by FACE

CS and hyaluronan (HA) levels in lung tissue were analyzed by FACE, as previously described [18, 20], with a modification to remove genomic DNA. Briefly, fetal lung tissue (~ 1 g) was micro-dissected to remove all visible blood vessels and airways using a dissecting microscope [21] and then GAGs were extracted as previously described [10]. Following extraction, the GAGs were resuspended in 100 mM ammonium acetate, pH 7.0, and the concentration of sulphated GAGs was measured by the 1,9-dimethylmethylene blue assay [22] using CS C as a standard (shark cartilage, Sigma-Aldrich). Aliquots containing 5 μg sulphated GAG were digested in ammonium acetate buffer (pH 7.0) with 10 mU of chondroitinase ABC (Seikagaku, Japan) for 16 h at 37 °C. The cooled samples were then centrifuged through MicroCon YM-3 filter devices, and the disaccharides recovered in the filtrate and freeze-dried. Freshly prepared 2-aminoacridone (5 μl; Bioscientific; 25 mg/ml dissolved in dimethylsulfoxide-acetic acid 85:15 vol/vol) was added to the dried disaccharides, vortexed, and incubated for 15 min at room temperature. Sodium cyanoborohydride (1 M, 5 μl) was then added to each tube, and fluorotagging occured at 37 °C for 18–20 h. The samples were cooled to room temperature and mixed with 10 μl of 37.5% glycerol, and 3-5 μl aliquots were analyzed immediately by electrophoresis on Mono Composite gels (Glyko or Epitope Technologies) or stored at − 70 °C. To enable quantification, monosaccharide standards of known picomolar amounts were fluorotagged for each experiment and analyzed on the same gels. Fluorescently tagged samples and standards were imaged and quantitated using Quantity One software (BioRad).

### Data analysis

All data are presented as a mean ± standard deviation (SD). Statistical analysis of data was performed using the Student’s unpaired t-test or one-way analysis of variance (ANOVA), as appropriate. Significant differences detected by the ANOVA, were subjected to the Fisher Least Significant Difference (LSD) post-hoc test to determine differences between individual group means. Statistical significance was taken at the *p* < 0.05 level.

## Results

### Fetal plasma cortisol concentrations

In cortisol-infused fetuses, plasma cortisol concentrations increased gradually over the infusion period from 7.6 ± 3.3 ng/mL before the start of the infusion to 34.3 ± 11.6 ng/mL on the last day of infusion (131d GA). Fetal plasma concentrations in saline-infused fetuses (2.3 ± 1.1 ng/mL) did not change during the experimental period [16]. Bilateral fetal ADX successfully abolished the pre-parturient increase in fetal plasma cortisol concentrations, resulting in a mean circulating concentration of ~ 2.0 ng/mL throughout the experimental period. In sham-operated control fetuses, circulating plasma cortisol concentrations remained unchanged from 120d GA (1.7 ± 0.1 ng/mL) until 135d GA (3.1 ± 0.7 ng/mL) but significantly increased to 11.4 ± 2.1 ng/mL at 140d GA and increased further to 19.6 ± 4.1 ng/mL at 143d GA; labour is expected at ~ 147 days of gestation in this breed of sheep.

### Fractional tissue volume

At 131d of GA (when the tissue was collected) the percentage of space occupied by peri-alveolar tissue was significantly lower in cortisol-infused fetuses (29.4 ± 0.9%) compared with saline-infused fetuses (33.0 ± 0.5%; *p* = 0.008, Fig. 1a). Similarly, fractional peri-alveolar tissue volumes were significantly reduced in 124d GA fetuses exposed to betamethasone (28.5 ± 0.2%) when compared to fetuses from saline-treated ewes (39.2 ± 0.7%; *p* < 0.0001, Fig. 1b). In contrast, the percentage of space occupied by lung tissue in bilateral ADX fetuses (33.8 ± 2.3%) at 143d GA tended to be greater than in sham-operated fetuses (28.3 ± 1.7%; Fig. 1c), however this failed to reach statistical significance (*p* = 0.08).

### Versican mRNA levels in fetal lung tissue

**Fig. 2:**
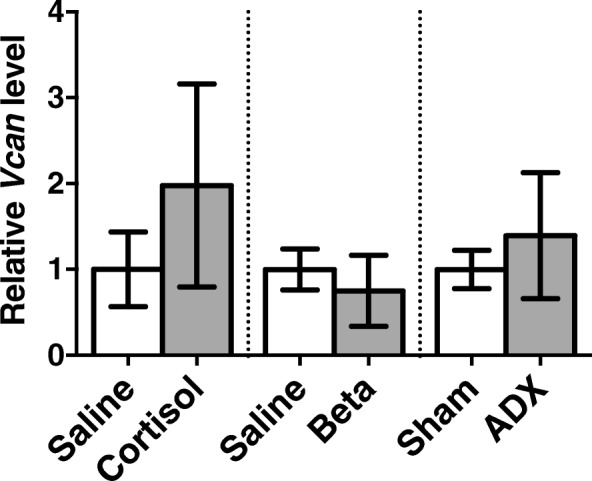
Mean ± SD Vcan mRNA levels (V0 plus V1 isoforms) in lung tissue collected from the left lung of control fetuses (131d saline-infused, n = 5; 124d antenatal saline-treatment, *n* = 6; 143d sham operated, n = 6) and fetuses exposed to cortisol-infusion, (131d n = 6), antenatal betamethasone treatment (Beta, 124d, n = 6) or bilaterally adrenalectomised (ADX, 143d, n = 6)

There was no differences in Vcan mRNA levels between cortisol-infused fetuses (2.0 ± 0.5) and saline-infused control fetuses (1.0 ± 0.2), between betamethasone treated fetuses (0.75 ± 0.17) and controls (1.0 ± 0.1), or between ADX fetuses (1.4 ± 0.3) and sham operated controls (1.0 ±0.1; Fig. 2).

### Versican deposition

**Fig. 3:**
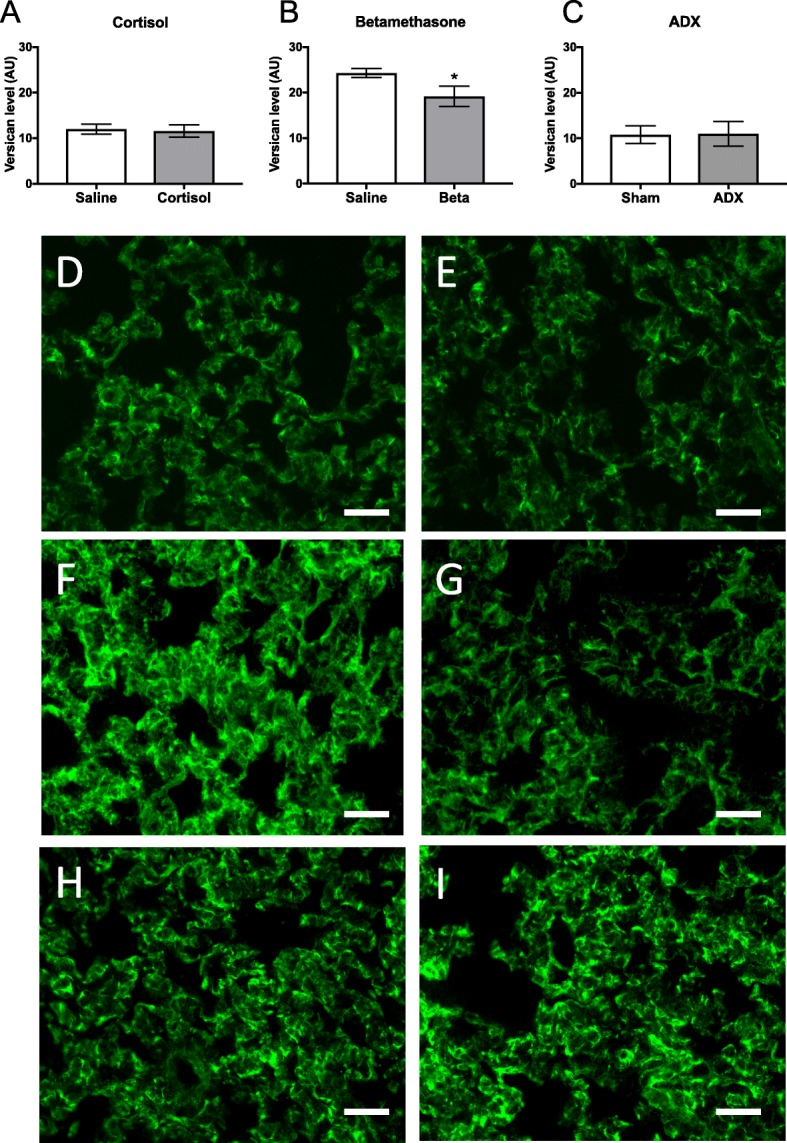
Mean ± SD versican deposition levels (per unit volume of lung tissue; arbitrary units, AU) in the peri-alveolar region of the lung measured in (**a**) cortisol-treated (n = 6) and saline-treated (n = 5), (**b**) betamethasone treated (n = 6) and saline-treated (n = 6), and (**c**) ADX (n = 6) and sham operated (n = 6) sheep lungs. Representative images of versican immunolocalisation (green) in lungs from (**d**) saline and (**e**) cortisol-treated sheep, (**f**) saline and (**g**) betamethasone-treated sheep, and (**h**) sham and (**i**) ADX-operated sheep. *p < 0.05 compared to controls. Scale = 25μm

Per unit volume of lung tissue, versican levels were similar in cortisol-infused (11.6 ± 0.6 AU) and saline-infused fetuses (12.0 ± 0.5 AU; Fig. 3a) and were similar in adrenalectomised (11.0 ± 1.1 AU) and sham-operated control fetuses (10.8 ± 0.8 AU; Fig. 3c). In contrast, versican levels were significantly decreased in fetuses from betamethasone-treated ewes (to 19.2 ± 0.9 AU) when compared with fetuses from saline-treated ewes (24.3 ± 0.4 AU; *p* = 0.0004; Fig. 3b).

### Chondrotin-4-sulphate (C-4-S) and Chondroitin-6-sulphate (C-6-S) deposition

#### C-4-S levels

**Fig. 4:**
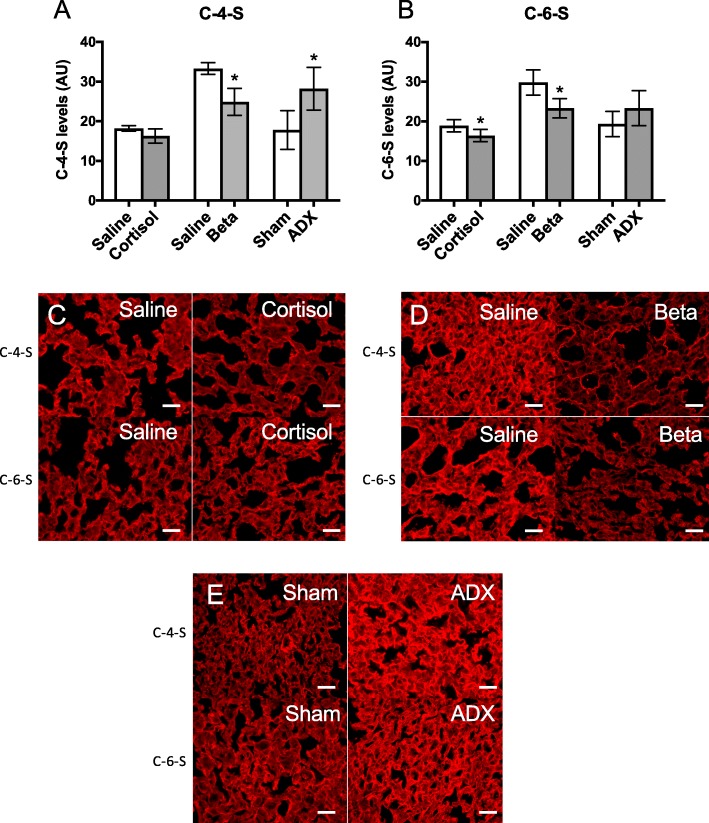
Mean ± SD (**a**) C-4-S and (**b**) C-6-S levels (per unit volume of lung tissue; arbitrary units, AU) in the peri-alveolar region of the lung measured in control fetuses (131d saline-infused, *n* = 5; 124d antenatal saline-treatment, *n* = 6; 143d sham operated, n = 6) and fetuses exposed to cortisol-infusion, (131d n = 6), antenatal betamethasone treatment (Beta , 124d, n = 6) or bilaterally adrenalectomised (ADX, 143d, n = 6). Immunolocalisation of C-4-S and C-6-S in the perialveolar region of lungs from (**c**) saline and cortisol-treated sheep, (**d**) saline and betamethasone-treated sheep, and (**e**) sham and ADX-operated sheep. *p < 0.05 compared to controls. Scale = 25μm

Per unit lung tissue volume, the level of C-4-S within the lung tended to be reduced in cortisol-infused fetuses (16.3 ± 0.8 AU) compared with saline-infused control fetuses (18.2 ± 0.3 AU; *p* = 0.06; Fig. 4a), however this failed to reach statistical significance. Bilateral ADX significantly increased C-4-S levels to 28.2 ± 2.2 AU compared with sham-operated control fetuses (17.8 ± 2.0 AU; *p* = 0.006; Fig. 4a) whereas betamethasone significantly decreased the level of C-4-S in lung tissue to 24.9 ± 1.4 AU compared with saline-treated control fetuses (33.3 ± 0.6 AU; *p* = 0.0003; Fig. 4a).

#### C-6-S levels

Per unit lung tissue volume, the level of C-6-S within lung tissue of cortisol-infused fetuses was decreased to 16.4 ± 0.7 AU compared to saline-infused control fetuses (18.9 ± 0.7 AU; *p* = 0.04, Fig. 4b). C-6-S levels in lung tissue was not different between ADX fetuses (23.3 ± 1.8 AU) compared to sham-operated control fetuses (19.3 ± 1.3 AU; Fig. 4b). In contrast, following betamethasone treatment, the level of C-6-S in lung tissue was reduced to 23.2 ± 1.0 AU compared to values in saline-treated control fetuses (29.8 ± 1.3 AU; *p* = 0.03; Fig. 4b).

### Sulphation profile of CS disaccharides

**Fig. 5:**
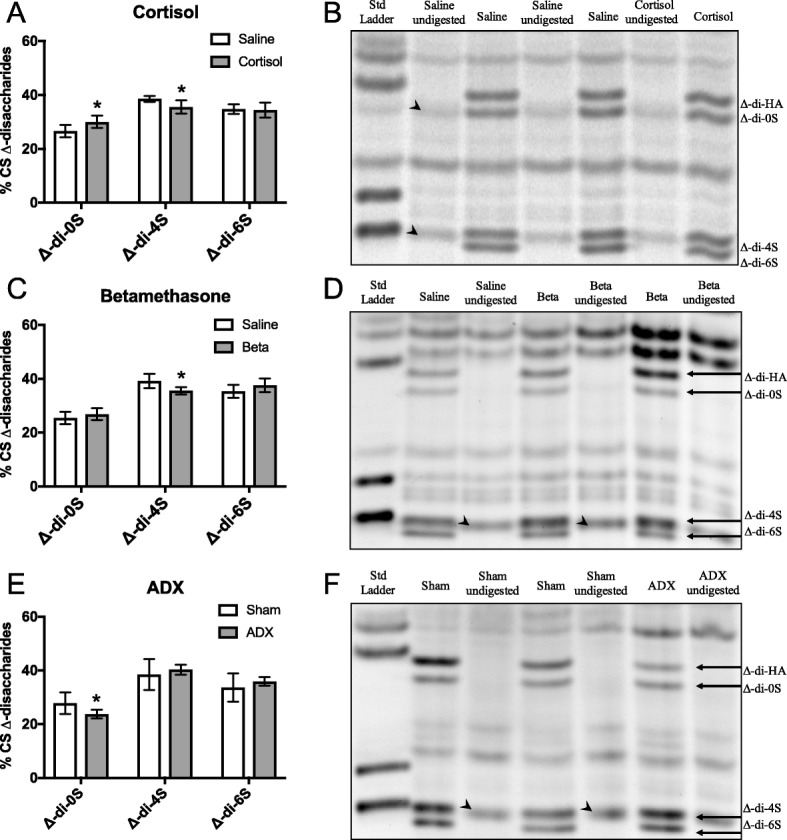
Mean ± SD proportions (percent of mono-sulphated and non-sulphated [CS] disaccharides) of the mono-sulphated (∆-di-4S and ∆-di-6S) and unsulphated (∆-di-0S) CS disaccharides measured in micro-dissected fetal lung tissue from fetuses (**a**) exposed to cortisol-infusion (131d n = 6), (**c**) exposed to antenatal betamethasone treatment (Beta, 124d, n = 6) or (**e**) bilaterally adrenalectomised (ADX, 143d, n = 6) and their relevant control fetuses. All values were measured by FACE analysis FACE gels depicting the sulphation profile of CS Δ-disaccharides in the lungs of fetuses (**b**) exposed to cortisol-infusion, (**d**) exposed to antenatal betamethasone treatment or (**f**) bilaterally adrenalectomised and their relevant control fetuses. A control sample without chondroitinase ABC digestion (labelled undigested) was electrophoresed alongside each chondroitinase ABC-digested sample in order to identify non-specific bands (arrow-heads). The density of non-specific background bands (migrating at the same position as specific bands) were substracted from the density of the bands of interest

The relative proportions of mono-sulphated (∆-di-4S and ∆-di-6S) and non-sulphated (∆-di-0S) disaccharides that comprised the chondroitin sulphate (CS) glycosaminoglycans isolated from fetal lung tissue were detected by FACE analysis; non-sulphated hyaluronan disaccharide units (∆-di-HA) were also detected. The percentage of non-sulphated ∆-di-0S was significantly increased to 30.0 ± 1% in cortisol treated fetuses compared with saline-infused fetuses (26.6 ± 1.0%; *p* = 0.04) whereas the percentage of mono-sulphated ∆-di-4S was significantly lower (35.5 ± 1.1%) in cortisol-treated fetuses compared to saline-treated fetuses (38.6 ± 0.5%; p = 0.03); the level of mono-sulphated ∆-di-6S remained unchanged (saline: 34.8 ± 0.8% vs. cortisol: 34.4 ± 1.2%; Fig. 5a,b).

The percentage of ∆-di-4S was decreased to 35.6 ± 0.6% in betamethasone-exposed fetuses compared to control fetuses (39.2 ± 1.2%; *p* = 0.02), whereas no changes could be detected in the percentage of ∆-di-0S (*saline:* 25.5 ± 1.0% vs. *betamethasone:* 26.9 ± 1.0%) or ∆-di-6S (*saline:* 35.3 ± 1.1% vs. *betamethasone:* 37.6 ± 1.1%; Fig. 5c,d).

The percentage of ∆-di-0S was lower in ADX fetuses (23.8 ± 0.8%) compared to sham-operated fetuses (27.8 ± 1.8%; *p* = 0.04) whereas, the percentage of mono-sulphated CS disaccharides, ∆-di-4S (40.3 ± 0.9%) and ∆-di-6S (35.9 ± 0.8%) in ADX fetuses were unchanged in comparison to sham-operated controls (38.5 ± 2.6% and 33.7 ± 2.4%, respectively; Fig. 5e,f).

### Hyaluronan levels

**Fig. 6:**
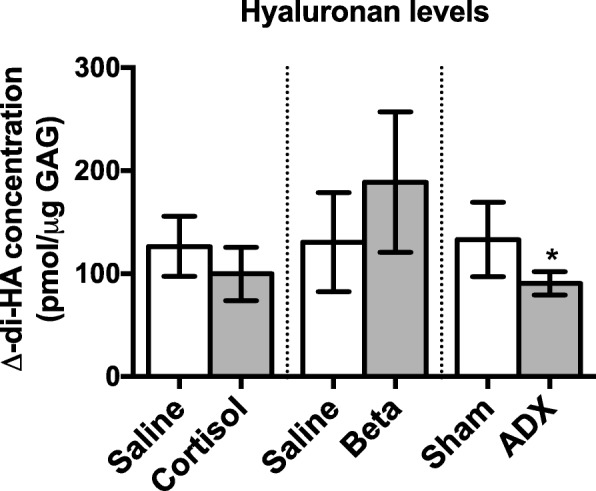
Mean ± SD HA levels (presented as pmol/μg GAG ± SD) in the peri-alveolar region of the fetal lung in control fetuses (131d saline-infused, *n* = 5; 124d antenatal saline-treatment, *n* = 6; 143d sham operated, *n* = 6) and fetuses exposed to cortisol-infusion, (131d *n* = 6), antenatal betamethasone treatment (Beta, 124d, *n* = 6) or bilaterally adrenalectomised (ADX, 143d, *n* = 6). All values were measured by FACE analysis and asterisks denote a significant difference between each treatment group (cortisol, ADX or betamethasone) and its relevant control group

Hyaluronan (HA) levels in the lung were similar in cortisol-infused fetuses (150.5 ± 18.4 pmol/μg GAG) and saline-infused fetuses (190.3 ± 22.8 pmol/μg GAG; Fig. 6, *p* < 0.05), and between betamethasone exposed fetuses (228.8 ± 35.2 pmol/mg) and saline-treated controls (130.7 ± 17.9 pmol/μg GAG). HA levels in the lung were significantly decreased in ADX fetuses (to 134.1 ± 6.8 pmol/μg GAG) compared to sham-operated control fetuses (177.8 ± 24.7 pmol/μg GAG; *p* = 0.02; Fig. 6).

### Cell proliferation levels

The percentage of proliferating lung cells following fetal ADX (1.69 ± 0.39%) was significantly higher than in sham-operated controls (0.64 ± 0.22%; *p* = 0.04; data not shown). There was no difference in the percentage of proliferating lung cells between maternal betamethasone-treated fetuses (0.86 ± 0.17%) and saline-treated controls (1.14 ± 0.12%; data not shown). We have previously reported that proliferation rates (measured via DNA synthesis rates) are not different following a 9-day fetal cortisol infusion compared to controls [23].

## Discussion

During fetal development, exogenous glucocorticoids have a potent stimulatory effect on fetal lung maturation, greatly reducing the incidence of respiratory distress in very preterm infants [24]. Although the precise underlying mechanisms are unknown, exogenous glucocorticoids are known to markedly reduce peri-alveolar tissue volumes, which involves changes in cell proliferation and remodelling of the ECM [13]. Versican is one of the most abundant CS-rich proteoglycans in the peri-alveolar region of the fetal lung [10]. Its high anionic charge density promotes water retention which contributes to tissue volumes and the viscoelastic properties of lung tissue. Versican levels in the lung decrease in the lead up to birth in mice [25] and sheep [10] and are closely associated with the normal developmental reduction in peri-alveolar tissue volumes [10]. Consequently, in this study we hypothesised that glucocorticoid-induced remodelling of the peri-alveolar interstitial tissue is mediated by changes in versican levels and/or to changes in the microstructure of CS GAGs. We found that cortisol administration to the fetus, which prematurely increased circulating cortisol concentrations in a manner similar to the normal pre-parturient increase in cortisol, induced changes in the sulphation level and microstructure of CS chains without changing versican levels. Antenatal betamethasone treatment, similar to that used clinically, induced much larger changes in the sulphation level and microstructure of CS chains and reduced versican levels within the peri-alveolar region of the lung.

Versican is widely distributed throughout the interstitial tissue compartment of the terminal airways, however, the finding that versican levels are not altered in response to increases and reductions in circulating cortisol levels (cortisol and ADX experiments) is consistent with our previous findings [10]. We have previously shown that versican levels markedly decrease between 90 and 126 days GA, but do not change between 126d and 138d GA, despite the increase in endogenous cortisol concentrations at this time [10]. Removal of the endogenous source of fetal cortisol (via ADX) failed to affect fetal lung Vcan mRNA levels and versican protein levels. Fetal cortisol infusion and maternal-betamethasone treatment also failed to alter Vcan mRNA levels, although betamethasone significantly reduced versican levels in parallel with the reduction with lung tissue volume. These results indicate that endogenous glucocorticoids are not a major regulator of fetal lung versican levels in sheep. This is in contrast to our previous studies, where we observed increased Vcan expression in mice that have the glucocorticoid receptor deleted from mesenchymal cells [11]. This suggests that loss of GR signalling prevents the normal decrease in versican in late gestation. It’s possible that the differences in the timing and duration of the removal of endogenous glucocorticoid signalling between the mouse and sheep experiments explains the contradictory results. Alternatively, cortisol may not be a major regulator of the normal developmental decrease in peri-alveolar tissue volumes, at least in fetal sheep despite the finding that adrenalectomy partially attenuated the reduction in lung tissue volumes observed at 143d GA. Indeed, the normal gestational-age related decrease in peri-alveolar tissue volumes and versican levels occurs much earlier than the pre-parturient increase in circulating cortisol levels [10].

In contrast to the cortisol and ADX treatments, which alter the levels of endogenous glucocorticoids, the levels of versican protein detected by immunofluorescence were reduced in the fetal lungs following maternal betamethasone-treatment. The differential response could be due to a dose-related effect, as the bioactivity of betamethasone is ~ 25 times greater than cortisol [26]. This suggests that glucocorticoid-induced changes in versican levels require a threshold of stimulation to be reached in the fetal sheep lung. Synthetic glucocorticoids induce greater tissue thinning than endogenous glucocorticoids in neonatal rat lungs [27], which could be due in part to differential regulation of versican levels. As many very preterm infants receive antenatal glucocorticoids, it is important to understand the precise mechanisms by which glucocorticoids affect lung development, as the developmental response might not be normal. Synthetic glucocorticoids are known to induce deleterious changes in the developing rat lung, such as reduced alveolarisation [28, 29] in addition to the known beneficial outcomes. Although the betamethasone-induced reduction in versican levels might lead to reduced air/blood gas barriers, thereby increasing the efficiency of gas transfer, alterations to the visco-elastic properties of lung tissue might make it more susceptible to volutrauma and shear stress injury, increasing the risk of bronchopulmonary dysplasia [24].

We have previously demonstrated a strong positive correlation between versican and HA levels during gestation [10]. However, in betamethasone-exposed fetuses, the decrease in versican was accompanied by a tendency for HA levels to increase. HA functions as an anchor for hyalectacan proteoglycans (such as versican) in interstitial tissue and binds versican to form HA-versican aggregates. Thus, an increase in HA towards term and following betamethasone exposure increases the potential to retain proteoglycans within the extracellular lung tissue compartment. However, the formation of stable HA-versican aggregates is dependent upon the presence of link proteins which are highly homologous to the G1 domain of proteoglycans such as versican [30]. Link proteins stabilise the interaction between proteoglycans and HA by locking proteoglycans into the HA chains, thereby anchoring them within the extracellular matrix [30]. Thus, the rapid decrease in versican levels and accompanying decrease in peri-alveolar tissue volumes in betamethasone-exposed fetuses might be associated with a reduction in link proteins. This would account for the apparent disconnection between versican and HA levels in lung tissue of these fetuses.

We have shown that endogenous and synthetic glucocorticoids affect the degree and pattern of sulphation on versican CS chains. Reduced sulphation of PGs reduces the CS charge density, thereby reducing the capacity to retain water within the lung tissue and maintain tissue volume. We found that both chondroitin-4-sulphate (C-4-S) and chondroitin-6-sulphate (C-6-S) levels are influenced by glucocorticoids, with betamethasone reducing both C-4-S and C-6-S. As versican is the primary CS PG in the lung and we have shown that C-4-S and C-6-S predominantly co-localise with versican [10], it is likely that the reduction in C-4-S and C-6-S levels in betamethasone treated sheep is due to the reduction in versican levels. However, cortisol infusion also decreased the level of C-6-S without altering versican levels, suggesting that glucocorticoids can reduce sulphation of versican in the fetal sheep lung. Conversely, ADX resulted in an increase in C-4-S density per unit tissue area without altering versican levels. Although the increase in C-4-S could be interpreted as an increase in sulphation, it is more likely that ADX (at ~112d GA) prevented the normal decrease in C-4-S that occurs late in gestation [10]. Furthermore, ADX may impede the normal structural maturation of the lung by maintaining sulphation levels of CS chains on versican and possibly other CS proteoglycans. Our data suggest that glucocorticoids reduce the sulphation of versican in the fetal sheep lung, which would result in reduced charge density and a reduced ability to maintain tissue volume in the lung, providing a possible mechanism for glucocorticoid-induced tissue thinning.

This mechanism is supported by our further analysis of the amount and proportion of non-sulphated and mono-sulphated CS disaccharides in peri-saccular/alveolar lung tissue (as assessed by FACE). Although this data indicated only minor changes in C-4-S and C-6-S, it is important to recognise that this analysis does not measure total content, but measures levels as a proportion of total sulfated GAG content (not including disulphated species), thereby explaining the discrepancy with the immunohistochemistry analysis. Unfortunately, this method did not allow us to quantify disulphated disaccharides and so the changes in the proportion of sulphated GAGs must be interpreted cautiously. Cortisol administration to the fetus increased the proportion of di-0S levels by 13% (expressed as a % total of non-sulphated and mono-sulphated GAGs), reduced the proportion of di-4S by 8% but did not affect the proportion of di-6S. These data demonstrate a small increase in non-sulphated CS disaccharides at the expense of mono-sulphated CS disaccharides, which must lead to an overall reduction in the net charge present on CS side chains. In contrast, ADX tended to decreased the proportion of di-0S (by ~ 14%) but did not alter the proportions of di-4S and di-6S compared to controls. Combined these data indicate that cortisol acts to decrease the net charge present on CS disaccharides leading to a reduction in anionic charge density. This would be expected to reduce the osmotic influence of PGs such as versican and may contribute to the decreased peri-alveolar tissue volumes associated with glucocorticoids.

It should be noted that in addition to maintaining tissue volume, versican also plays a role in regulating cell proliferation. Versican expression is often correlated with high cell proliferation rates, particularly in cancer [14] and has been shown to bind to the cell-cycle regulator Midkine [31]. Mice studies suggest that one of the major roles of glucocorticoids in the lung is reducing proliferation of interstitial cells late in development, leading to thinning of lung tissue [11–13]. The changes in versican levels and sulfation in our study do not reflect changes in proliferation in these models. We found a significant increase in the percentage of proliferating lung cells in ADX-treated fetuses, with no change in versican levels and a prevention of the normal decrease in sulphation late is gestation. In contrast there is no change in proliferation in the lungs of betamethasone-treated fetuses or cortisol-infused fetuses [23], even though versican levels and sulphation are altered in these treatment groups. The specific mechanism by which versican promotes proliferation is not fully understood and may be secondary to its role is promoting ECM remodelling. For example, in arterial smooth muscle cells, platelet derived growth factor increases Vcan expression, which increases the ECM expansion required for proliferation of these cells [32]. It is possible that versican does not directly regulate cell proliferation in the developing lungs and that the changes in Vcan expression in glucocorticoid receptor knockout mice reflect changes in ECM remodelling [11, 13]. Glucocorticoids may alter versican levels and sulphation in the developing lung, thereby altering ECM remodelling and then depending on the developmental timing and length of exposure/removal of glucocorticoids, the downstream effects of altered versican activity may differ.

We have previously shown that fetal sheep lung only expresses two isoforms of versican, V0 and V1 [10]. The V0 and V1 isoforms contain the largest domains for CS glycosaminoglycan attachment and are likely to have the greatest influence on tissue hydration. Fetal lung mRNA levels for versican were not altered by a cortisol infusion which is consistent with our previous finding that mRNA levels for V0 and V1 are constant over the latter half of gestation, despite increasing cortisol levels over this time [10]. Similarly, fetal lung versican mRNA levels were not affected by maternal betamethasone administration, suggesting that synthetic glucocorticoids do not reduce versican content by reducing its gene expression.

## Conclusion

In summary, we have shown that glucocorticoid regulation of versican is a complex process, with multiple aspects of versican synthesis, degradation and/or modification likely to be affected. Endogenous glucocorticoids, while not altering the level of versican itself, regulate the degree of sulphation of CS chains, which likely affects their ability to maintain tissue volume within the fetal lung. In contrast, the highly potent synthetic glucocorticoid, betamethasone, markedly reduces versican content, as well as the degree of sulphation of versican, within the fetal lung. These results are consistent with the suggestion that alterations in versican and the degree of sulphation of CS side chains play an important role in regulating peri-alveolar tissue volumes. Furthermore, these findings indicate that synthetic glucocorticoids do not simply mimic the effect of endogenous cortisol and may act on the fetal lung via different mechanisms.

## Abbreviations

∆-di-0S: Non-sulphated disaccharide
∆-di-4S and ∆-di-6S: Mono-sulphated disaccharides
AU: Arbitrary unit
BPD: Bronchopulmonary dysplasia
C-4-S: Chondrotin-4-sulphate
C-6-S: Chondroitin-6-sulphate
CS: Chondroitin sulphate
ECM: Extracellular matrix
FACE: Fluorophore assisted carbohydrate electrophoresis
GAG: Glycosaminoglycan
GalNAc: N-acetylgalactosamine
GlcUA: Glucuronic acid
HA: Hyaluronan
IHC: Immunohistochemistry
